# Exercise Pathophysiology and Testing in Individuals With a Fontan Circulation

**DOI:** 10.1016/j.cjcpc.2023.01.001

**Published:** 2023-01-14

**Authors:** Arjun K. Mahendran, David Katz, Alexander R. Opotowsky, Adam M. Lubert

**Affiliations:** Department of Pediatrics, Heart Institute, Cincinnati Children’s Hospital, University of Cincinnati College of Medicine, Cincinnati, Ohio, USA

## Abstract

The Fontan circulation, a surgical palliation for single-ventricle congenital heart disease, profoundly impacts the cardiopulmonary response to exercise. Reliant on passive pulmonary blood flow, the Fontan circulation has limited capacity to augment cardiac output as necessary to supply working muscles during exercise. Cardiopulmonary exercise testing (CPET) objectively assesses cardiorespiratory fitness and provides insight into the etiology of exercise intolerance. Furthermore, CPET variables, such as peak oxygen consumption and submaximal variables, have prognostic value and may be used as meaningful endpoints in research studies. CPET is also useful in clinical research applications to assess the effect of pharmacologic or other interventions. Medical therapies to improve exercise tolerance in individuals with a Fontan circulation, such as pulmonary vasodilators, may modestly improve peak oxygen consumption. Exercise training focused on aerobic fitness and lower extremity strength may have a more consistent and larger impact on these measures of aerobic fitness. CPET is a valuable diagnostic and prognostic tool for those with a Fontan circulation. Newer ancillary assessments, such as noninvasive peripheral venous pressure monitoring and cardiac output measurements, hold promise to provide a more nuanced insight into the underlying pathophysiology.

## Résumé

La circulation de Fontan, une solution chirurgicale palliative aux cardiopathies congénitales univentriculaires, a de profondes répercussions sur la réponse cardiorespiratoire à l’exercice physique. Puisqu’elle repose sur un flux sanguin pulmonaire passif, la circulation de Fontan présente une capacité limitée à accroître le débit cardiaque au besoin pour assurer l’approvisionnement des muscles durant l’effort physique. L’épreuve d’effort cardiorespiratoire (EECR) permet de mesurer la capacité cardiorespiratoire de manière objective et de mieux comprendre les causes de l’intolérance à l’effort. En outre, les variables mesurées lors de l’EECR, comme la consommation maximale d’oxygène (VO_2_) et les variables sous-maximales, ont une valeur pronostique et pourraient constituer des critères d’évaluation pertinents dans les travaux de recherche.

L’EECR possède également des applications utiles en recherche clinique pour évaluer les effets des interventions pharmacologiques ou d’autre nature. Les traitements médicaux qui visent à améliorer la tolérance à l’effort chez les personnes ayant une circulation de Fontan, comme les vasodilatateurs pulmonaires, pourraient entraîner une modeste augmentation de la valeur maximale de VO_2_. L’entraînement physique axé sur la capacité aérobique et sur la force des membres inférieurs pourrait avoir un effet plus constant et important sur ces mesures de la capacité aérobique. L’EECR est un précieux outil diagnostique et pronostique pour l’évaluation des patients ayant une circulation de Fontan. Des méthodes d’évaluation complémentaires plus récentes, comme la surveillance non invasive de la pression veineuse périphérique et les mesures du débit cardiaque, sont des avenues prometteuses vers une compréhension plus nuancée des mécanismes physiopathologiques sous-jacents.

**Figure 1:**
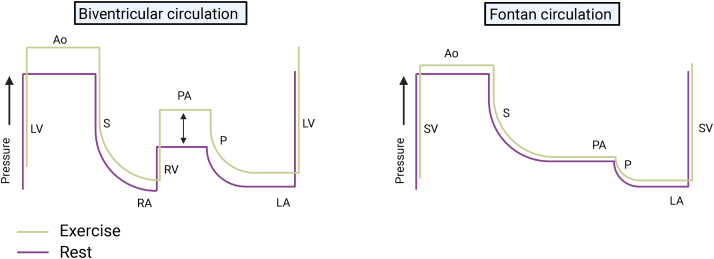
A schematic representation of systolic pressure through a biventricular circulation and Fontan circulation during rest and exercise. In a biventricular circulation, pressure is generated in the left ventricle (LV) and right ventricle (RV); that pressure is dissipated across the systemic microvasculature (S) and pulmonary microvasculature (P), respectively. With exercise, higher pressure (**black arrows**) is generated in both ventricles; that augmentation of power by the RV ensures that there is adequate LV preload without direct coupling to systemic venous pressure (or RA pressure). In contrast, in the Fontan circulation, it is the systemic ventricle (SV) that must generate pressure to propel blood through both the systemic and pulmonary circulations. With exercise, transpulmonary flow can only be augmented by modest contributions of the systemic muscle pump to augment venous return and reduction of pulmonary vascular resistance, implying markedly limited reserve. Lacking this, transpulmonary flow, and consequently left atrial (LA) blood return/preload, does not increase sufficiently and cardiac output cannot increase to meet the demands of exercise. Adapted from La Gerche and Gewillig.^3^

The Fontan procedure is often the ultimate operation in a series of palliative surgeries for individuals born with single-ventricle congenital heart disease. These surgeries result in a total cavopulmonary (or atriopulmonary) connection that is meant to volume unload the single ventricle and relieve cyanosis. To maintain cardiac output (CO), the Fontan circulation relies on venous pressure to passively drive systemic venous blood through the pulmonary vascular bed to the pulmonary venous atrium, given the absence of a subpulmonary ventricular pump.^1^ Chronically elevated systemic venous pressure, however, is associated with long-term adverse sequelae. That, along with the lack of a subpulmonary pump, intrinsically limits the ability to augment systemic ventricular preload (Fig. 1), which leads to a very high prevalence of impaired maximal aerobic exercise capacity (referred to throughout the article as “peak VO_2_”).^2^

At rest, the well-compensated Fontan circulation can provide a normal CO. The constraints of the Fontan circulation are exposed during exercise, where the demand for increasing oxygen delivery to working muscle exceeds the capacity to augment CO. This article provides a comprehensive review of the current understanding of the mechanisms of exercise limitation, conventional and novel aspects of cardiopulmonary exercise testing (CPET), prognostic implications of CPET, and the available therapies to improve maximal aerobic exercise capacity in individuals with a Fontan circulation. The current review focuses on the maximal aerobic exercise testing and does not explore other approaches that may be useful but are less commonly applied (eg, 6-minute walk test, timed up and go, muscle strength measurement).

## Factors Influencing Maximal Aerobic Exercise Capacity

**Figure 2:**
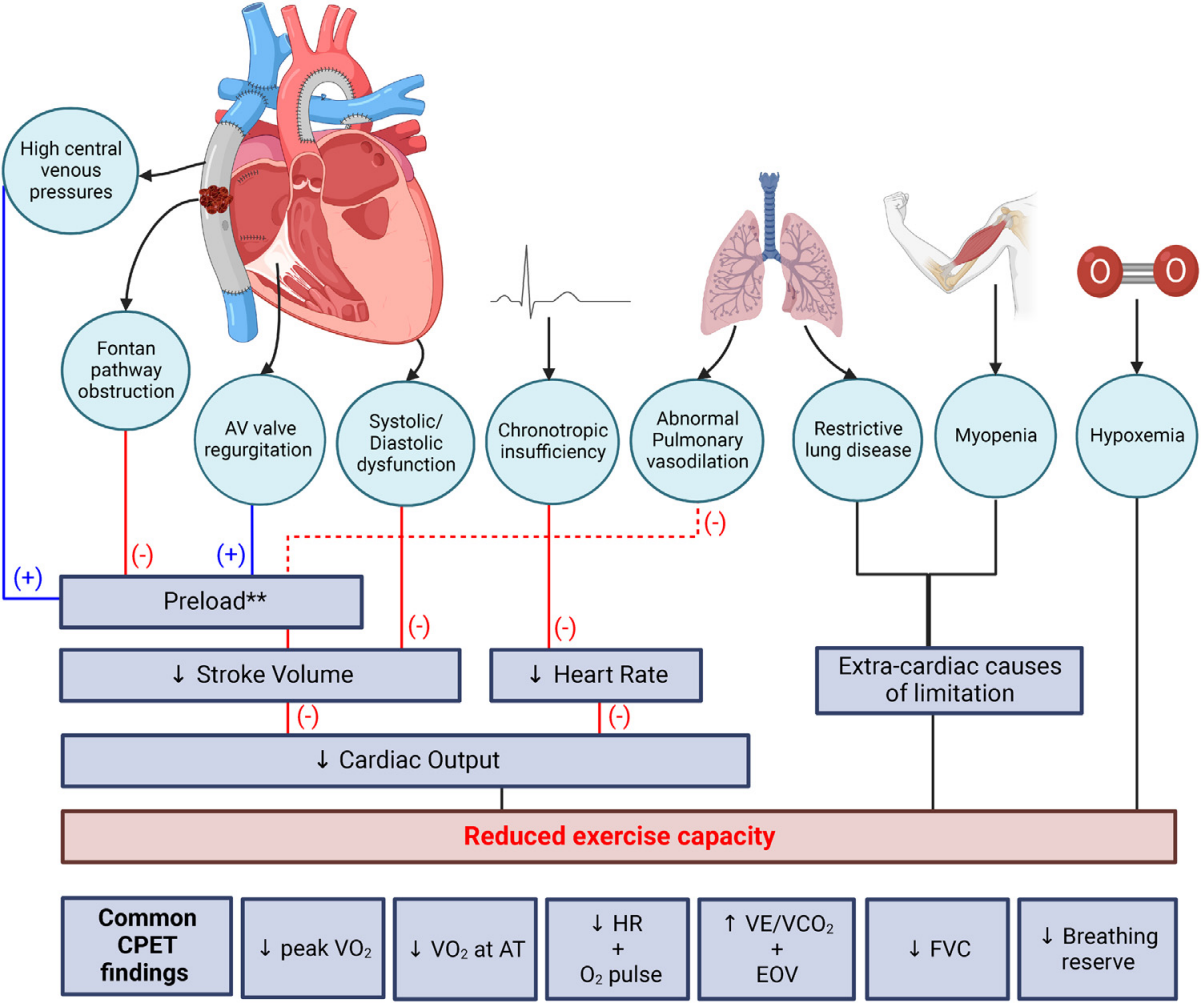
Reduced exercise capacity caused by multiple Fontan-associated physiological and haemodynamic abnormalities. Haemodynamic alterations after the Fontan operation are shown at the top of the diagram. Consequent alterations in exercise response are depicted at the bottom. ∗∗Preload is reduced by a variety of factors or increased to a point that it negatively affects the stroke volume. Illustration created using BioRender (San Francisco, California). AT, anaerobic threshold; AV, atrioventricular; EOV, exercise associated oscillatory ventilation; FVC = forced vital capacity; Ve/VCO_2_, minute ventilation/carbon dioxide production slope; VO_2_, oxygen consumption.

To perform physical work, oxygen delivery must be augmented to meet increased metabolic demands. Increasing CO is one of the fundamental ways in which oxygen delivery is augmented. In a biventricular circulation, the subpulmonary ventricle can support a robust increase in pulmonary blood flow without a large change in systemic venous pressure, and, subsequently, systemic ventricular preload, to augment stroke volume and CO. In a Fontan circulation, pulmonary blood flow and preload are directly coupled to central venous pressure, with dependence on the skeletal muscle pump, negative intrathoracic pressure, and low pulmonary vascular resistance. Collectively, these are not equivalent to a dedicated subpulmonary pump (Fig. 2).^3^ Although this review focuses on factors affecting oxygen delivery, increased metabolic demand is also partly satisfied by increased oxygen extraction from arterial blood. Furthermore, there are several Fontan-independent factors that affect maximal aerobic exercise capacity including biological sex, age, body composition, baseline fitness, and genetic predisposition; these are beyond the scope of this review.

### Fontan pathway and pulmonary arteries

Obstruction anywhere along the Fontan pathway may impair haemodynamic efficiency, increasing proximal venous pressure while impairing systemic venous return and limiting CO augmentation.^4^ Likewise, pulmonary artery stenosis and abnormal pulmonary artery flow distribution can also be associated with energy loss.^5^, ^6^, ^7^, ^8^ In addition to reducing CO, asymmetric pulmonary blood flow causes ventilation-perfusion mismatch, inefficient ventilation (increased minute ventilation [Ve]:carbon dioxide production [VCO_2_]), and increased work of breathing.

Fluid dynamic studies have demonstrated that the extracardiac Fontan is associated with less energy loss than the lateral tunnel Fontan, possibly due to circular rigid conduit and surgically created caval offset.^9^ However, extracardiac conduits are more likely to become stenotic,^10^ and conduit obstruction significantly impairs haemodynamic efficiency and, as a result, exercise performance.^11^^,^^12^ Early Fontan palliation may require that a small conduit be placed; with growth, this can be associated with increased impedance to venous return.^10^ Conversely, an oversized conduit may cause increased energy loss within the conduit, also impacting the ability to augment venous return and CO. Investigators have proposed heuristics for optimal Fontan conduit sizing to minimize energy loss and maximize exercise tolerance.^13^^,^^14^

### Pulmonary vascular bed

During exercise in the biventricular circulation, an increase in pulmonary blood flow recruits and dilates pulmonary arterioles and capillaries, increases pulmonary vascular cross-sectional area, and thus results in lowering pulmonary vascular resistance.^15^^,^^16^ Conversely, in the Fontan circulation, exercise is not associated with a consistent, substantial decline in pulmonary vascular resistance.^17^^,^^18^ In addition to the impaired augmentation of venous return and lack of pulsatility (ie, higher systolic pressure recruiting and distending vessels), this may be related to endothelial changes and dysfunction, likely related to chronic, nonpulsatile flow.^19^^,^^20^

### Lung mechanics

Reliant on low pulmonary vascular resistance, optimal Fontan haemodynamics depend on efficient lung mechanics. Abnormal lung mechanics impairs the negative intrathoracic pressure required to “pull” blood through the Fontan circulation. Restrictive lung disease is commonly seen in individuals with a Fontan circulation due to a variety of factors, including previous surgeries, intrinsic lung abnormalities, diaphragm paresis/paralysis, and pleural stiffness.^21^, ^22^, ^23^, ^24^ During exercise, restrictive lung disease is associated with reduced O_2_ pulse, higher Ve, and higher Ve/VCO_2_ slope. Diffusion capacity is reduced, often despite the preserved membrane component of diffusion capacity, possibly due to lower pulmonary capillary blood volume, which depends on position (ie, lower capillary volume in the upright position) in the absence of a subpulmonary pump.^23^^,^^25^

### Ventricular function

Advanced systolic dysfunction may limit CO in a subset of individuals with a Fontan circulation.^26^ Diastolic dysfunction is frequently appreciated, present in 35%-57% of individuals with a Fontan circulation, and its presence is associated with lower peak VO_2_ and supine peak work.^17^^,^^27^, ^28^, ^29^ Diastolic dysfunction is associated with higher central venous pressure and Fontan circulatory failure.^30^ Ultimately, the ventricle can only pump what is allowed by the Fontan circuit and is limited by preload.^1^ Preload is defined as myocardial stretch and is a function of filling pressure and ventricular compliance; lower ventricular compliance results in lower preload at the same filling pressure. The impact of diastolic dysfunction often manifests during exercise, with increased heart rate and reduced diastolic filling time.^30^ To uncover occult diastolic dysfunction, some centres perform fluid challenges during haemodynamic catheterization.^28^ However, it remains unclear whether a fluid challenge recapitulates day-to-day meaningful pathophysiology.

### Skeletal muscle and the muscle pump

Studies using bioimpedance and dual-energy x-ray absorptiometry have demonstrated that individuals with a Fontan circulation often have abnormal body composition. Compared with healthy peers, these individuals have reduced muscle mass, increased adiposity, and shorter stature.^31^^,^^32^ There is decreased skeletal muscle mass, particularly in the lower extremities, though the pathophysiology of low muscle mass is unclear.^32^ Without a subpulmonary ventricular pump, lower extremity muscle contraction is important to drive venous blood through the Fontan circuit and augment preload during exercise.^33^ Higher muscle mass is associated with greater CO augmentation during exercise and increased peak VO_2_.^32^^,^^34^^,^^35^ Although further research is necessary, exercises that maintain muscle mass, especially lower extremity muscle mass, should be encouraged.

## Exercise Testing Protocol for the Fontan Circulation

There is no universally superior or established testing protocol for those with a Fontan circulation. Included below is the testing protocol used at our centre, which likely varies from other congenital heart centres based on the available equipment and resources. There is no established age to begin exercise testing, but in our institution, it usually begins at ages 10-12 years and repeated every 1-2 years. We favour using a maximal ramp cycle ergometer protocol. Although the disadvantages of cycle ergometry include lower peak VO_2_ and earlier lower extremity muscle fatigue, there are substantial advantages compared with a treadmill test including measurable work rate and less motion-related artefact, allowing for cleaner, more interpretable data.^36^ A ramp protocol allows for a continuously increasing work rate rather than other exercise protocols that have larger increments in workload. This allows for a more accurate assessment of peak VO_2_.^37^ Alternatively, submaximal protocols such as the modified Bruce treadmill protocol or the Astrand-Rhyming cycle protocol can be used if submaximal indices are of interest. Although children as young as 6 years can perform CPET appropriately, special equipment such as a paediatric treadmill or cycle ergometer may be necessary to appropriately test paediatric patients.

Before the start of exercise, a baseline electrocardiogram and spirometry are performed. In addition, our CPET laboratory measures pulmonary blood flow (approximating CO in the absence of shunt) using an inert gas rebreathing method (Innocor CO).^38^^,^^39^

In some cases, we also place an 18-gauge peripheral intravenous catheter in an upper extremity vein, which is then used to monitor peripheral venous pressure during exercise (see the paper by Colman et al.^40^ for details of the protocol).

The rate of increase of the ramp cycle ergometry protocol is selected by the exercise physiologists based on the patient’s expected fitness, with a goal to achieve a maximal, symptom-limited test within approximately 6-12 minutes for children or adults. Electrocardiogram and pulse oximetry are continuously monitored, and gas rebreathing CO and vital signs are measured intermittently during exercise and recovery. Venous pressures are recorded at baseline, at each minute during exercise, at peak exercise, and during the recovery at 1, 3, and 5 minutes after peak exercise.

Pacemakers are not uncommon in individuals with a Fontan circulation due to a high incidence of sinus node dysfunction.^41^ Often, a rate-responsive pacemaker’s accelerometer is not adequately engaged during cycle ergometer exercise. Thus, the heart rate response generated during the exercise is inadequate, which affects the oxygen pulse (ratio of VO_2_ to heart rate) and CO. In this instance, we confirm if the pacemaker rate response is adequately optimized. If there is still an inadequate response, a treadmill ramp protocol may be used instead, which more consistently triggers the accelerometer and rate response.

Musculoskeletal and neurological impairments are also commonly seen in this population.^42^ With a careful assessment of the patient’s baseline functioning, an appropriate exercise testing modality is selected. A cycle ergometer often allows adequate exercise testing despite mild motor deficits.^36^ In those with such impairments, a cycle ergometer can also provide a safer option.

## Alterations in Exercise Response in the Fontan Circulation

### Peak workload

Table 1Peak workload is the maximal workload or power output (work/time) achieved by the patient during an exercise test. For exercise tests using a cycle ergometer, the power output is directly measured in watts. For exercise tests using a treadmill, workload can be estimated using equations incorporating treadmill speed, grade of incline, and body mass; however, such estimates are often inaccurate.^36^ Peak workload is reduced in those with a Fontan circulation compared with the general population, with data reporting ranges from 47% ± 21%^43^ of predicted to 91% ± 32%.^44^ Exercise training programmes can improve peak workload.^45^

**Table 1:** Overview of cardiopulmonary exercise testing (CPET) variables and findings commonly observed in individuals with a Fontan circulation.

CPET variable	Description	Comparison to healthy peers		Comments
*Peak workload (% predicted)*^44^^,^^45^	Maximal power output produced.	↓	47%-91% predicted	Workload only measurable using a cycle ergometer; not measured by a treadmill.
*Peak VO*_*2*_*(% predicted)*^49^, ^50^, ^51^, ^52^, ^53^, ^54^	Maximal amount of oxygen consumed (used) during exercise; used as a surrogate of cardiac output.	↓	Mean approximately 55%-60% predicted	Associated with outcomes. Often used as an endpoint in research studies. A minority have normal peak VO_2_ (approximately 10%-33%).
*VO*_*2*_*at AT (% predicted)*^62^^,^^63^	Oxygen consumption during sub-maximal exercise, at the ventilatory anaerobic threshold.	↔	Approximately 80 % predicted	Gradual decline compared with peak VO_2_. Has prognostic value.
*Peak O*_*2*_*pulse (% predicted)**^54^*	Ratio of oxygen consumption to heart rate; reflects amount of oxygen ejected from ventricle in each cardiac cycle.	↓	50%-70% predicted	A surrogate of stroke volume, assuming normal haemoglobin concentration and oxygen extraction.
*Peak heart rate (bpm)*^27^^,^^44^^,^^64^	Maximal heart rate achieved during exercise.	↓	65%-75% predicted	Both peak HR and HR recovery have prognostic value.
*Ve/VCO*_*2*_*slope*^23^^,^^50^	Linear relationship of the volume of air breathed out and the amount of carbon dioxide produced throughout exercise.	↑	Usually elevated	Normal < 30, though varies by age. Most have mild elevation, though may be moderately or more elevated.
*Forced* v*ital* c*apacity (litres*L*)*^22^, ^23^, ^24^, ^25^	Amount of air that can be forcibly exhaled from the lungs after taking the deepest breath possible.	↓	Approximately 45% have FVC less than the lower limit of normal	May have multiple causes (e.g., abnormal pulmonary development; prior surgeries).
*Breathing reserve (BR) (% of MVV)*^43^^,^^72^	Difference between the volume of air breathed during rest and the maximum breathing capacity.	↓	Low BR present in approximately 22%	Reduced BR (< 20%-30% of MVV) suggests a pulmonary contribution to exercise limitation. Often presents a secondary limitation.
*Exercise oscillatory ventilation (EOV)**^74^*	Regular, oscillatory, and periodic breathing pattern during exercise.	↑	Present in approximately 37%	Presence independently predicts death or transplantation.
*Oxygen saturation (%)*^43^^,^^48^^,^^65^^,^^68^	Systemic arterial oxygen saturation measured during exercise, usually with non-invasive pulse oximetry.	Nl or ↓	Mean peak O_2_ saturation approximately 88%-92%	Reduced O_2_ saturation may indicate a right-left shunt, but may be due to other causes (e.g., parenchymal lung disease).

FVC, forced vital capacity; HR, heart rate; MVV, maximal voluntary ventilation; Ve/VCO_2_, minute ventilation/carbon dioxide production slope; VO_2_, oxygen consumption; VO_2_at AT, oxygen consumption at ventilatory anaerobic threshold.

### Peak VO_2_

Peak VO_2_ is the maximal oxygen consumption a patient achieves during an exercise test. It is often used as a surrogate for the more conceptually pure VO_2_ max, as most individuals with a Fontan circulation are not able to achieve a true VO_2_ plateau. VO_2_ max, or peak VO_2_, is often considered to be a surrogate for peak CO; this is generally regarded as the single most important indicator of cardiopulmonary function in adults and children with congenital heart disease.^46^^,^^47^ VO_2_ is affected by any process that impairs oxygen delivery or VO_2_, including impaired muscle oxygen extraction, decreased CO (chronotropic incompetence, decreased stroke volume), or systemic arterial hypoxaemia. Peak VO_2_ is often reduced in individuals with a Fontan circulation and, on average, is 55%-60% of what would be predicted for the general population.^48^, ^49^, ^50^, ^51^, ^52^, ^53^ Compared with other congenital heart diseases, the Fontan circulation has one of the lowest median percent predicted VO_2_, ahead of only Eisenmenger syndrome and other complex cyanotic heart diseases.^53^ Although reduced peak VO_2_ is the norm, approximately 10%-33% of Fontan patients have normal or even supranormal peak VO_2_.^54^^,^^55^

There is, however, a progressive decline in VO_2_ relative to the general population during adolescence; this decline occurs to a lesser extent after achieving adult size. It more closely parallels the slow decline in peak VO_2_ seen after the mid-twenties in the general population but with lower absolute VO_2_ values.^52^^,^^56^^,^^57^

Those with a morphologic systemic left ventricle may, on average, have higher VO_2_ than those with systemic right ventricle, though individual differences are substantial.^58^ Earlier age at the time of Fontan completion may also be related to higher peak VO_2_ at late follow-up.^56^^,^^59^ It is not certain whether the type of Fontan affects maximum aerobic exercise capacity. Direct comparison is challenging because other aspects of care have evolved along with the change in the preferred Fontan approach; for example, few patients with hypoplastic left heart syndrome will have had an atriopulmonary Fontan. Empirical comparisons have given rise to variable findings.^56^^,^^60^

### VO_2_ at the ventilatory anaerobic threshold

As above, most individuals with a Fontan circulation have a peak VO_2_ below the normal range of their biventricular peers. However, during submaximal exercise, such as at the ventilatory anaerobic threshold (VO_2_ at AT), the limitations of Fontan physiology have not yet been exceeded. That is, the Fontan circulation is still able to augment CO. Although still reduced compared with healthy controls, VO_2_ at AT is better preserved relative to population norms than peak VO_2_.^61^^,^^62^ Abnormalities in VO_2_ at AT may provide different insights into function and prognosis, with some suggesting this as a preferable endpoint because most day-to-day activity is performed at or below the AT.^61^

### Heart rate

Chronotropic impairment, often defined simply as an inability to reach at least 85% of maximal predicted heart rate, is common regardless of the ventricular morphology and type of Fontan. Both reduced peak heart rate and elevated resting heart rate are commonly observed compared with healthy peers.^26^^,^^43^^,^^63^ Furthermore, heart rate recovery after exercise is impaired, with one study reporting 154% longer than healthy controls.^64^ Peak heart rate achieved also decreases over time at a similar rate to healthy controls.^65^ Although sinus node dysfunction is common, from another perspective, one might suggest that the heart rate response may be appropriate for the exercise intensity and CO (ie, the heart rate could increase further but exercise has to stop because of other reasons). Thus, the inadequacy of heart rate response may be more reflective of the underlying abnormal haemodynamics rather than true sinoatrial dysfunction.^66^ This seems logical, but we should question whether this association supports the implied inference; in most circumstances, when stroke volume augmentation is inadequate for circumstance, there is a compensatory increase in sympathetic tone and, with a normally functioning sinus node, this causes increased heart rate. By way of comparison, in the biventricular circulation, exercise in the upright position compared with the supine position is associated with similar CO, but with a lower stroke volume and higher heart rate.^67^ This is intuitive, and we rightly expect a more accentuated heart rate response in the setting of impaired stroke volume augmentation, not the reverse.

### Oxygen saturation

One of the goals of single-ventricle palliation is to normalize systemic oxygen saturation. However, oxygen saturation remains reduced after the Fontan palliation and can be attributed to intrapulmonary right-to-left shunting, persistent Fontan fenestration, coronary sinus return to the pulmonary venous chamber, and venovenous collaterals. With exercise, oxygen saturation decreases with a mean nadir O_2_ saturation of approximately 89% at peak exercise.^43^^,^^48^^,^^65^^,^^68^ There is also abnormal differential pulmonary blood flow to the different lung zones with an increased flow to the upper lobes. The resulting V/Q mismatch may also exacerbate hypoxaemia.^69^ A surgical Fontan fenestration created at the time of Fontan completion is often performed to maintain CO, at the expense of right-to-left shunt and hypoxaemia. With favourable Fontan haemodynamics, subsequent fenestration device closure may increase peak VO_2_, with less hypoxaemia and lower Ve/VCO_2_ slope.^70^^,^^71^

### Ventilatory responses

Breathing reserve is the difference between the maximal ventilation at peak exercise and the maximum voluntary ventilation (normal value approximately 30%-40% of maximal voluntary ventilation; this variable can also be presented as an absolute value in litres per minute). Approximately one-fifth of individuals with a Fontan circulation have a reduced breathing reserve, suggesting a pulmonary limitation.^43^^,^^72^

The relationship between Ve and VCO_2_, measured either as a ratio at a specific time point (eg, Ve/VCO_2_ at anaerobic threshold or at nadir) or as a slope (eg, Ve/VCO_2_ slope) below the respiratory compensation point is a dimensionless ratio that indicates how many litres of air is needed to be breathed in to eliminate a litre of CO_2_. Normally, the Ve/VCO_2_ slope is <30 (though the upper end of the normal range modestly increases with aging), and a higher slope is associated with poor outcomes in a variety of cardiovascular conditions.^36^ Individuals with a Fontan circulation have a higher Ve/VCO_2_ slope than the general population; this is for a number of distinct reasons. There is often some degree of persistent right-to-left shunt; there may be a higher prevalence of pulmonary vascular disease interfering with diffusion, and there is ventilatory perfusion mismatch in the setting of nonpulsatile blood flow.^22^^,^^49^

A phenomenon called exercise oscillatory ventilation (EOV), defined by a periodic breathing pattern during exercise with oscillatory Ve persisting for >60% of the duration of the exercise test, is also commonly seen (up to 37.5%) in individuals with a Fontan circulation. Its presence is independently associated with adverse outcomes.^73^ Both Ve/VCO_2_ slope and EOV can often be assessed on a submaximal test (respiratory expiratory ratio <1.1).

### Systemic venous pressure response to exercise

In the presence of impaired pulmonary vasodilation during exercise, central venous pressure increases with exercise as venous return to the central circulation increases. As central and peripheral venous pressures are closely approximated, a noninvasive method that is gaining popularity is peripheral venous pressure measurement, which can be used as a surrogate to approximate central venous pressure changes during exercise.^74^ Higher peripheral venous pressure (eg, >25 mm Hg) at peak exercise in the Fontan circulation suggests a static or dynamic obstruction in the Fontan pathway and is independently associated with adverse cardiovascular outcomes.^40^ More comprehensive invasive exercise testing may have a role in distinguishing underlying causes of Fontan circulatory limitation, such as diastolic dysfunction and elevated pulmonary vascular resistance, during exercise that is not otherwise apparent.^75^^,^^76^

## Exercise Physiology in Children and Adults

There are several differences between paediatric and adult responses to exercise. These differences should be considered when evaluating studies involving populations with a wide age range. Compared with adults, children have a smaller stroke volume and compensate by generating a higher heart rate during exercise. In children and adolescents, the maximal heart rate approximates 195-200 bpm.^77^ Despite the higher heart rate, CO (and thus peak VO_2_) is lower in children than adults and increases with age. Hormonal changes during childhood also affect exercise physiology with postpubertal males having a higher arteriovenous oxygen difference than that of postpubertal females.^78^ This is related to age- and sex-related increases in haemoglobin and skeletal muscle mass.^79^ With expected age-related increases in CO, one can expect any haemodynamic alterations that negatively affect CO to have increased prognostic value in adults compared with children. When compared with adults, children are less capable of recruiting high-threshold, type II muscle fibres.^80^ Children, therefore, have a lower maximal power output but greater muscle endurance and quicker recovery from high-intensity, short-term exercise. This has implications when designing paediatric exercise training protocols.

## Using Exercise Testing as a “Surrogate” Cardiovascular Endpoint

CPET may also be used as a surrogate cardiovascular endpoint, with VO_2_ commonly the main variable of focus. Both peak VO_2_ and change in peak VO_2_ over time are associated with the likelihood of adverse cardiovascular outcomes and mortality. Fernandes et al.^48^ reported that peak VO_2_ <16.6 mL/kg/min was associated with substantially higher mortality (hazard ratio [HR]: 7.5, 95% confidence interval [CI]: 2.6-21.6). Another study reported that peak VO_2_ <21.0 mL/kg/min independently predicted mortality.^68^ A decrease in peak VO_2_ of >3% per year was associated with an increased 5-year risk of adverse cardiovascular events.^49^ Another study showed that a reduction in VO_2_ by 10% was a predictor of death or transplant (HR: 2.5, CI: 1.5-4.2, *P* < 0.01).^52^

Submaximal measures such as VO_2_ at AT can be used for prognostication, particularly useful in the context of a submaximal test. Lower VO_2_ at AT is associated with a greater risk of death or transplant; one study identified a cutoff value of <9 mL/kg/min associated with increased mortality (HR: 5.5; CI: 2.1-14.8).^48^^,^^50^ Clinical trials often use peak VO_2_ as a primary outcome measure, but some investigators have proposed using VO_2_ at AT as an outcome measure instead. Submaximal exertion may be more representative of the daily activities of individuals with Fontan physiology. Another argument is that, by peak exercise, the central venous pressure has hit a physiological limit and may not be amenable to improvement.^81^ Identifying the anaerobic threshold, however, may be associated with substantial error and uncertainty. The fundamental premise that peak VO_2_ should not be considered the definitive endpoint is valid whatever the merits of VO_2_ at AT. Other submaximal exercise variables, such as the oxygen uptake efficiency curve (logarithmic relationship between VO_2_ and Ve), may also provide prognostic information.^82^

CPET variables other than peak VO_2_ have also been shown to be associated with cardiovascular outcomes. Peak heart rate and heart rate reserve (difference between resting and maximal heart rate) are strongly associated with mortality risk and increased cardiac hospitalization.^48^^,^^49^^,^^68^ A study showed that a peak heart rate <122 bpm was a risk factor with increased mortality risk.^48^ Heart rate reserve was an independent risk factor to predict death or transplant as well as hospitalization rate.^51^ The presence of EOV is independently associated with death or transplantation (HR: 3.9; CI: 1.5-10.0).^73^ Although the elevated Ve/VCO_2_ slope predicts worse outcomes in biventricular heart failure, there are mixed data on the relationship between elevated Ve/VCO_2_ and adverse outcomes in individuals with a Fontan circulation.^48^^,^^51^^,^^68^^,^^82^^,^^83^ This likely relates to the many causes of higher Ve/VCO_2_, as detailed above. Finally, impaired pulmonary function (reduced forced vital capacity) has also been identified as an independent predictor of mortality.^22^

## Clinical Applications for Exercise Testing

CPET in individual with a Fontan circulation provides fundamental insights on the level of fitness, specific cardiopulmonary impairments to exercise, and also identifies patients at high risk of adverse outcomes. Repeated, regular CPET provides additive valuable data on clinical trajectory. Serial, objective measurement of exercise response is a cornerstone of the clinical assessment of the Fontan circulation, dependent on performing reproducible, standardized CPET. One of the most clinically useful findings is an abrupt, meaningful decrease in exercise tolerance (eg, a substantial decrease in peak VO_2_ or exercise duration). This should trigger a comprehensive assessment of why this may have occurred, with further testing (eg, cardiac catheterization) and intervention on residual lesions (eg, valve repair or relief of Fontan pathway obstruction). Even in the absence of apparent reversible cause, this information can provide strong motivation for exercise training and may imply more urgency to begin evaluation for transplant. Furthermore, in symptomatic individuals, follow-up exercise testing after a trial of a new medication may aid in decisions about whether to continue therapy or pivot to a new approach. Although there are no data to guide optimal frequency of testing, we would recommend relatively frequent testing on the order of every 1-2 years for adolescents and adults to ensure timely identification of a clinical change. This frequency is in line with the American Heart Association scientific statement on Fontan management.^84^

## Therapies to Improve Maximal Aerobic Exercise Capacity

### Medical therapies

Pulmonary vasodilator therapy has been investigated for those with a Fontan circulation because they would be expected to lower pulmonary vascular resistance and thereby improve preload to the systemic ventricle.

The TEMPO trial (Treatment with Endothelin Receptor Antagonist in Fontan Patients) evaluated the impact of bosentan, an endothelin receptor blocker, on peak VO_2_ in 75 adolescents and adults randomized 1:1 to bosentan or placebo.^85^ After 14 weeks, peak VO_2_ increased by 2 mL/kg/min in the bosentan group compared with an increase of 0.6 mL/kg/min in the control group (*P* = 0.02). Furthermore, 9 patients in the bosentan group had improvement in functional class (eg, from NYHA class II to I) compared with none of those in the placebo arm. However, a larger trial involving macitentan showed no significant improvement in the primary outcome of peak VO_2_.^86^

The FUEL trial (Fontan Udenafil Exercise Longitudinal), a multicentred, randomized controlled trial, examined the effect of udenafil, a phosphodiesterase type 5 inhibitor, on peak VO_2_.^81^ There was no significant impact on the primary outcome; peak VO_2_ increased by 2.8% in the udenafil group compared with a reduction of 0.2% in the placebo group (*P* for difference = 0.07). There were improvements in submaximal exercise variables and markers of ventricular function.

Compared with oral or intravenous administration, inhaled administration would be expected to more selectively decrease pulmonary vascular resistance. An acute treatment trial using a more selective pulmonary vasodilator, inhaled iloprost (a prostacyclin analog), showed an increase in oxygen pulse and peak VO_2_, especially in patients with peak VO_2_ <30 mL/kg/min.^87^

Other smaller studies of phosphodiesterase type 5 inhibitors and endothelial receptor antagonists have reported mixed results on peak VO_2_ or data at submaximal exercise.^88^, ^89^, ^90^, ^91^, ^92^ A meta-analysis concluded that pulmonary vasodilator therapy is likely safe but that there is no appreciable significant benefit in terms of cardiorespiratory fitness, pulmonary vascular resistance, or quality of life.^93^

Importantly, randomized clinical trials of these therapies have all been performed in cohorts of clinically stable patients, generally those with baseline good functional status. Although there have been anecdotal reports of those experiencing deterioration, systematic investigation is lacking. The data overall on pulmonary vasodilators suggest a neutral or modest increase in peak VO_2_ of questionable clinical significance in generally well individuals. There is no evidence to date that pulmonary vasodilators have any long-term benefit. That said, it remains plausible that the concomitant reduction in venous pressure may deliver a long-term benefit that is yet to be explored. It is unclear, however, in what context an improvement in maximal aerobic exercise capacity would be a reason to start a medication, independent of any improvement in symptoms or outcomes.

### Exercise training

Many individuals with a Fontan circulation are sedentary.^94^ Exercise training is safe and effective in improving fitness in a variety of congenital heart diseases.^95^ A recent systematic review of exercise training for individuals with a Fontan circulation demonstrated improvement in peak VO_2_ in the majority of the included studies involving different exercise modalities (aerobic, resistance training, and inspiratory muscle training).^96^ With supervised exercise programmes, there was a higher rate of VO_2_ improvement compared with those with unsupervised home programmes (75% vs 38%). There was a lower rate of improvement with higher baseline VO_2_ with maximal benefit seen in those with lower baseline VO_2_. This suggests that exercise programmes improve an individual’s underlying deconditioned state, rather than improving Fontan haemodynamics. Another review focusing on paediatric (<20 years old) exercise programmes showed that at least 1 measure of exercise capacity improved with exercise training programmes.^97^

A small investigational study of 11 patients studied the impact of high-weight lower extremity resistance training.^98^ The investigators hypothesized that lower extremity muscle mass would augment the peripheral muscle pump in individuals with a Fontan circulation, who lack a subpulmonary pump. After training, participants had significantly increased muscle mass, strength, and peak VO_2_. Furthermore, with detraining, not only did muscle mass decrease, but so did peak workload, VO_2_, and oxygen pulse. Children and adults have similar strength gains with resistance training. However, strength gain in children is not associated with muscle hypertrophy, and children respond better to a high-repetition, moderate resistance protocol.^80^

Studies have also explored the effects of inspiratory muscle training using resistance-based breathing exercises to strengthen respiratory muscles.^99^^,^^100^ One study reported that although inspiratory training improved inspiratory muscle strength and ventilatory efficiency (lowered Ve/VCO_2_ slope), it did not improve peak VO_2_.^100^ Inspiratory muscle training also improved CO at rest but did not improve exercise CO. This may imply that the negative intrathoracic pressure created by inspiration has greater impact on pulmonary blood flow at rest compared with during exercise.^101^

There is a general consensus that regular physical activity should be encouraged in the Fontan population; not only is physical activity associated with higher peak VO_2_, and possibly with better prognosis, but it is also associated with improved quality of life.^102^ Patients who self-report an active lifestyle were more likely to have preserved VO_2_.^54^ Those who engage in consistent physical activity experienced some improvement in their peak VO_2_ during their early adult years.^103^ Physical activity may also have a protective effect on the liver.^54^ Although evidence remains limited, given the low concern for adverse consequences and potential for real benefit, we strongly encourage exercise training in this population with a specific focus on lower extremity resistance training. Regardless of the intervention, however, maintaining a habit of regular, at least moderately intense physical activity and exercise remains a challenge for many, if not most, individuals.^104^

## Conclusions

Measuring maximal aerobic exercise capacity and more subtle characteristics of exercise response with CPET in individuals with a Fontan circulation provides valuable diagnostic, mechanistic, and prognostic information. An appropriately performed and interpreted CPET, accounting for striking differences in exercise response in the Fontan circulation compared with the biventricular circulation, can assess the degree of exercise limitation and the primary causes of that limitation, as well as suggesting why a given patient may be experiencing symptoms.
